# Parathyroidectomy Resolves Tooth Discoloration: A New Presenting Sign of Hyperparathyroidism?

**DOI:** 10.1155/2020/8281468

**Published:** 2020-05-11

**Authors:** Estella Musacchio, Andrea Piotto, Pierluigi Binotto, Antonio Toniato, Leonardo Sartori

**Affiliations:** ^1^Department of Medicine DIMED-Clinica Medica 1-University of Padova, Via Giustiniani 2, 35128 Padova, Italy; ^2^Department of Surgical, Oncological, and Gastroenterological Sciences-Clinica Chirurgica 3-University of Padova, Via Giustiniani 2, 35128 Padova, Italy; ^3^Private Dental Practice, Via M. Polo 10, 35123 Padova, Italy; ^4^Endocrine Surgery Unit, Istituto Oncologico Veneto IOV-IRCCS, Via Gattamelata 64, 35128 Padova, Italy

## Abstract

**Introduction:**

We report the resolution of tooth discoloration following parathyroidectomy in an otherwise asymptomatic woman with primary hyperparathyroidism-associated hypercalcemia. *Case Report*. A 59-year-old Caucasian woman, diagnosed with primary hyperparathyroidism in 2011, nonsmoker with excellent overall oral health. She complained of tooth discoloration starting in 2013. Pigmentation was particularly evident in the necks of the lower central and lateral incisors (Vita Classical score C2). No bleaching was undertaken. Parathyroidectomy was performed five years after primary hyperparathyroidism diagnosis. Six months later, a reduction in pigmentation was strikingly evident, with incisors scoring A1 and A2. The improvement persisted over time. Tooth value also increased compared to baseline.

**Conclusions:**

This is, to our knowledge, the first report that parathyroidectomy might resolve dental discoloration. This outcome deserves investigation in a meaningful sample size and may eventually prompt the inclusion of dental issues among the consequences of primary hyperparathyroidism.

## 1. Introduction

Primary hyperparathyroidism (PHPT) is a systemic disease that affects 0.2-1.0% of the general population. Its prevalence is higher in subjects > 65 yrs and in postmenopausal women [1, 2].

The diagnosis of PHPT often is made by occasionally finding elevated serum calcium levels, confirmed by elevated levels of PTH. The treatment of choice for PHPT is parathyroidectomy (PTx) indicated not only in symptomatic patients but also in asymptomatic ones with hypercalcemia, hypercalciuria, and reduced bone density, according to recent guidelines [3, 4]. As a metabolic bone disease, PHPT affects the entire skeleton and may cause abnormalities in the oral cavity, likely by inducing alterations in oral hard tissues or by exacerbating common pathological processes [5].

While most reports about the effects of PTH on dental structures concern experimental models [6, 7], literature regarding oral manifestation of PHPT and/or related biochemical alterations is limited [8, 9]. A very recent systematic review of oral manifestations related to HPT identifies a wide variation in the presentation of the disease concluding that bone pathology, most commonly reported in literature, should not be assumed as the only oral sequelae of HPT [10].

We here report the interesting case of the resolution of tooth pigmentation following PTx in an otherwise asymptomatic woman with PHPT-associated hypercalcemia. This outcome has been reported by patients referred to our clinic but never addressed before on a formal scientific basis. We believe that it may be overlooked by surgeons and endocrinologists despite its frequent occurrence.

## 2. Case Report

This is a case report of a nonsmoker Caucasian woman, age 59. Routine biochemical laboratory testing done at menopause in 2010 showed hypercalcemia, 24 hrs hypercalciuria, and elevated PTH. US scan identified a solitary parathyroid adenoma of the upper right gland (12 × 3 mm). The patient was diagnosed with PHPT in 2011. ^99m^Tc-sestamibi scintigraphy for ectopic sites was negative. PTH, calcemia, ionized calcium, and 24 hrs calciuria were over the upper limit at baseline, two months prior to surgery. Serum and urinary phosphorous, 25-OH vitamin D, serum albumin, eGFR, and all other biochemical parameters were within the normal ranges. Levels were evaluated preoperatively and monitored every month for the first 6 months and thereafter at 12 months (Table 1).

### 2.1. Medical History

Since age 15, the patient underwent several orthopedic surgeries for traumatic lesions of the ligaments. In all cases, the recovery was complete. Osteopenic at both the lumbar spine and the femur, she underwent i.v. bisphosphonates (zoledronic acid 5 mg) twice, once at 22 months and once at 4 months before PTx, to slow down the HPT-associated bone loss. Home medications included cholecalciferol (7500 UI weekly), antihypertensive therapy, and occasional analgesics.

### 2.2. Oral Status

The patient had 27 teeth. All four third molars were extracted at age 23 for prophylactic reasons. The second lower left molar was extracted following a trauma at age 24. The patient had no prostheses or implants, and her general oral health was excellent except for gingival recession of the front teeth, more evident in the lower arch. She never smoked nor took any medication known to interfere with dental pigmentation and regularly underwent oral hygiene treatments at 6- to 8-month intervals. She also presented with one right torus mandibularis of 6 mm in length and 4 mm in elevation. The patient had no sialolythiasis. Light microscope analysis of her saliva showed no crystal deposits of any kind. Beginning in 2013, the patient complained of tooth discoloration, particularly evident in the necks of the lower central and lateral incisors. No bleaching was performed.

Tooth shade was evaluated by visual assessment independently by three calibrated examiners (one dentist and two oral hygienists) using a shade guide (Vita Classical A1-D4) [11]. Range covers 16 tooth shades from white to reddish-grey: A1-A4 (reddish-brownish), B1-B4 (reddish-yellowish), C1-C4 (greyish shades), and D2-D4 (reddish-grey). Assessments were repeated during follow-ups (at 1, 6, and 12 months), each performed in the same room, at the same time of the day and under the same artificial central and positional lights. Disagreements were discussed and resolved by a fourth professional, a dentist. Dental pigmentation tended toward green-grey shades in the front teeth and was particularly evident at the neck level of the front incisors where it was C2. Posterior teeth such as molars and premolars did not exhibit the same pronounced pattern, possibly because there was no gingival recession, so the most pigmented area was not visible. Tooth value, a subjective index of the relative brightness or darkness, was negative [12, 13].

### 2.3. Outcome

For personal reasons, as PHPT was not extremely severe, the patient preferred to delay PTx: it was eventually performed six years after the first finding of hypercalcemia. Histology was positive for chief cell adenoma arranged in solid, trabecular, and pseudoacinar patterns. After surgery, the therapy was integrated for one month with calcitriol 25 *μ*g twice daily and calcium carbonate 1000 mg, in order to prevent secondary HPT following calcium drop. Calcium carbonate was substituted with calcium citrate and finally discontinued due to intolerance. Dietary calcium-containing food allowed a total calcium intake of 500-800 mg daily. One month after PTx, cholecalciferol supplements were resumed according to the preoperative schedule.

The patient's main characteristics before and after endocrine surgery are reported in the table. Biochemical profile was characterized by higher serum calcium and elevated PTH levels. PTH dropped from 130 to 13 ng/L intraoperatively, but increased afterwards, reaching 64.3 ng/L one year after surgery. At a subsequent evaluation at 18 months, PTH declined to 48.2 ng/L (data not shown).

Six months after PTx, the reversal of dental discoloration was strikingly evident with a score of A1 and A2 for the upper and the lower incisors, respectively. This persisted throughout the remaining follow-up period and was recently confirmed at the latest check-up. Tooth value also increased compared to baseline. Dental sensitivity, self-reported by the patient as mild, did not change.

## 3. Discussion

PTx was accompanied by a dramatic decrease in tooth discoloration and by an improvement in value that paralleled the decrease observed in total and ionized serum calcium levels but was independent of HPT resolution. Indeed, the patient showed a prompt decline of hypercalcemia, while PTH remained over the upper limit, even if at a lower extent compared to the levels recorded before surgery. Such persistence of higher PTH must be considered secondary to the low serum calcium levels. Visible calcium deposition in hypercalcemic states is well described with respect to the eye and also the tooth (in vitamin D intoxication), but changes in teeth color were never reported before [14]. Providing a rationale to this finding is problematic. The tooth being the most calcified structure of the body, a tropism, or selective calcium deposition can be hypothesized with the meaning of storage/scavenger of excess calcium ions at the dentin level, less calcified but more metabolically active than the enamel and therefore more prone to subsequent calcium ion release [15, 16]. This proposition could also explain why the color variation happened so sudden. The actual molecular mechanism needs to be clarified.

Padbury et al. reported a higher frequency of tori in a population affected with PHPT [5], as a consequence of an imbalance between cortical and trabecular bone turnover. The pathogenesis of tori has long been debated and is generally thought to be multifactorial with contributions of genetic as well as environmental factors, such as mandibular shape and occlusal forces [17]. Our patient did not show any reduction in torus size over the follow-up period, nor did substantial changes occur in femoral and lumbar BMD. Note that PTH levels persisted over the upper limit. Also, the times of bone remodeling are quite long so that, in general, tori disappearance may be as slow as their growth.

Today's profile of PHPT manifestations in the oral cavity is more subtle than what was described decades ago [10]. To our knowledge, tooth pigmentation has never been reported as a potential sign. We are aware that tooth-shade assessment would be more objective if carried out with a shade-matching electronic instrument such as the one used in prosthetic restoration. However, in the presented case, the goal was not aesthetic and the measures conducted under controlled and reproducible conditions by healthcare professionals are reliable enough to adequately describe the finding [18], particularly since the shade differences before and after surgery were remarkable.

As we have now entered the “era of outward appearance,” both the physical and aesthetic perfections of the body have never been pursued so widely. With all the media and social pressure, much emphasis is also devoted to the harmony and regularity of the mouth and teeth whitening has become a major issue in a person's self-approval [19]. Given these premises, we believe that the oral professionals' role in recognizing unacknowledged PHPT patients may be relevant. In cases of subjects with refractory or recurrent tooth discoloration despite hygiene and whitening sessions, PHPT may be suspected. Checking PTH and calcemia levels would be a simple, cost-effective, and valuable procedure borne of the kind of cooperation among healthcare specialists that should be a basis of public health strategies [20].

## 4. Conclusions

HPT uncovering could exemplify how different medical branches should interact for the overall benefit of the patient. Consequences of untreated PHPT may be serious and lead to clinically significant complications, with a subsequent economic burden. Nevertheless, a clear marker of the disease is missing and HPT diagnosis is often casual.

The present finding is to our knowledge the first report of dental pigmentation resolution after PTx. We believe it deserves deeper investigation with a larger sample size and may eventually prompt the inclusion of dental issues among the consequences and possible markers of PHPT.

## Figures and Tables

**Table 1:** Patient's characteristics at baseline and follow-up.

Variable	Normal range	At PHPT diagnosis^†^	Baseline^‡^	FU 1 month	FU 6 months	FU 12 months
Age (yrs)		54	59	—	—	60
*Biochemistry*						
PTH (ng/L)	6.5-36.8	58.2	72.0	48.3	49.9	64.3
Ca total (mmol/L)^§^	2.10-2.55	2.58	2.76	2.32	2.43	2.37
Ca^2+^ (mmol/L)^§§^	1.19-1.29	1.36	1.36	1.18	1.15	1.19
u-Ca (mmol/24 h)	2.5-7.5	8.62	8.62	na	na	3.93
p-P (mmol/L)	0.87-1.45	0.91	0.82	na	1.04	0.97
u-P (mmol/24 h)	12.9-42	15.6	19.5	na	na	17.0
25-OH vitamin D (nmol/L)	>75	78	82	na	na	87
*Bone mineral density (BMD)*						
Lumbar spine L2-L4 *T* score (SD)		-1.6	-1.8	na	na	-1.9
Total femur *T* score (SD)		-1.4	-2.0	na	na	-2.0
*Oral assessment*						
Teeth (*n*)		27	27	27	27	27
Front upper incisors (vita scale)		A2	C2	C2	A2	A2
Lateral upper incisors (vita scale)		A1	C1	C1	A2-A1	A1
Front lower incisors (vita scale)		A2	C2	C2	A2	A2
Lateral lower incisors (vita scale)		A2	C2	C2	A2	A2
Torus elevation (mm)		na	4	4	4	4

PHPT = primary hyperparathyroidism; FU = follow-up; na = not available/not assessed. ^†^PHPT diagnosis was made 5 yrs prior to surgery. ^‡^Baseline assessment was performed two months prior to surgery. ^§^Total serum calcium, not adjusted for albumin. ^§§^Ionized calcium, collected and measured according to the current recommendations.

## Data Availability

The data set underlying the findings in our study is within the paper. The original clinical records are in the archives of the Padova University Hospital.
